# Efficacy of Utilization of All-Plant-Based and Commercial Low-Fishmeal Feeds in Two Divergently Selected Strains of Rainbow Trout (*Oncorhynchus mykiss*): Focus on Growth Performance, Whole-Body Proximate Composition, and Intestinal Microbiome

**DOI:** 10.3389/fphys.2022.892550

**Published:** 2022-05-20

**Authors:** Ilaria Biasato, Simona Rimoldi, Christian Caimi, Sara Bellezza Oddon, Giulia Chemello, Marino Prearo, Marco Saroglia, Ronald Hardy, Laura Gasco, Genciana Terova

**Affiliations:** ^1^ Department of Agricultural, Forest and Food Sciences, University of Turin, Grugliasco (TO), Italy; ^2^ Department of Biotechnology and Life Sciences, University of Insubria, Varese, Italy; ^3^ Department of Life and Environmental Sciences, Marche Polytechnic University, Ancona, Italy; ^4^ The Veterinary Medical Research Institute for Piedmont, Liguria and Aosta Valley, Torino, Italy; ^5^ Hagerman Fish Culture Experiment Station, University of Idaho, Hagerman, United States

**Keywords:** rainbow trout, selective breeding, performance, vegetable proteins, gut microbiome

## Abstract

The present study aimed to investigate the growth performance, whole-body proximate composition, and intestinal microbiome of rainbow trout strains when selected and non-selected for weight gain on all-plant protein diets. A 2x2 factorial design was applied, where a selected (United States) and a non-selected (ITA) rainbow trout strain were fed using either an all-plant protein (PP) or a commercial low-FM diet (C). Diets were fed to five replicates of 20 (PP) or 25 (C) fish for 105 days. At the end of the trial, growth parameters were assessed, and whole fish (15 pools of three fish/diet) and gut samples (six fish/diet) were collected for whole-body proximate composition and gut microbiome analyses, respectively. Independent of the administered diet, the United States strain showed higher survival, final body weight, weight gain, and specific growth rate when compared to the ITA fish (*p* < 0.001). Furthermore, decreased whole-body ether extract content was identified in the PP-fed United States rainbow trout when compared to the ITA strain fed the same diet (*p* < 0.001). Gut microbiome analysis revealed the *Cetobacterium* probiotic-like genus as clearly associated with the United States rainbow trout, along with the up-regulation of the pathway involved in starch and sucrose metabolism. In summary, the overall improvement in growth performance and, to a lesser extent, whole-body proximate composition observed in the selected rainbow trout strain was accompanied by specific, positive modulation of the intestinal microbiome.

## Introduction

Genetic selection has progressively gained a foothold in the aquaculture sector, as it allows obtaining individuals with desired phenotypic traits. Traditional selective breeding—where only fish that exhibit desirable characteristics for one or multiple traits are chosen and bred—represents the most commonly adopted genetic enhancement technique. It is mainly directed towards the maximization of growth potential, improvement in fillet quality attributes, or development of resistance to stress and pathogens (Xu et al., 2015). In particular, rainbow trout (*Oncorhynchus mykiss*) has frequently been the object of selective breeding, with current programs being predominantly based on mass selection for growth rates (Cleveland et al., 2020) and resistance to viral or bacterial diseases (Vallejo et al., 2020; Zuo et al., 2020). Among all the selective breeding programs carried out to improve the growth potential of rainbow trout, one of the most impactful is related to the efficiency of digesting all-plant protein feeds (Overturf et al., 2013; Lazzarotto et al., 2015; Callet et al., 2017). It is, indeed, well known that the aquaculture sector has been trying to overcome sustainability challenges for the past decade, as worldwide production from aquaculture has grown rapidly and overtaken capture fisheries, and per capita consumption of fish has doubled (FAO, 2020). Therefore, the unsustainability of a “fish-to-fish” feeding has progressively made it necessary to replace fishmeal (FM) by alternative protein sources of plant origin in diets for farming fish. However, all-plant protein feeds do not represent the optimal choice for salmonids, with reductions in feed intake, weight gain, feed efficiency, alteration of gut microflora and immune response, and intestinal dysfunction and inflammation (mainly affecting the distal gut) being commonly observed (Krogdahl et al., 2010). In the light of such negative outcomes, genetic selection has successfully proved to be an effective counteracting measure to create rainbow trout strains that outperform the commercial, parental lines in utilizing all-plant protein diets without showing worsened growth performance or developing distal enteritis (Burr et al., 2012; Abernathy et al., 2017; Callet et al., 2017).

So far, the efficacy of selective breeding for utilization of all-plant protein diets in rainbow trout has been investigated by comparing selected and non-selected strains when fed either an FM or an all-plant protein diet in terms of growth response, nutrient retention, plasma amino acid (AA) patterns, and gut, liver, and muscle transcriptomic profiles (Abernathy et al., 2017; Callet et al., 2017; Brezas and Hardy, 2020; Brezas et al., 2021; Callet et al., 2021). In particular, candidate genes for protein (i.e., intestinal AA transporters and ribosomal proteins), lipid (i.e., long-chain polyunsaturated fatty acids biosynthesis), and purine–thiamine metabolisms, as well as energy production and inflammation (i.e., interleukin receptors), have previously been linked to synchronization of AA absorption and protein digestion rates and, in turn, improved growth and protein retention in the selected strains (Abernathy et al., 2017; Brezas et al., 2021; Callet et al., 2021). However, as gut microbiome alterations can influence fish performance (Vargas-Albores et al., 2021), and the intestinal microbiome itself may be, in turn, modulated by dietary changes (Rimoldi et al., 2018 and, 2021; Gaudioso et al., 2021; Terova et al., 2021), the characterization of the gut microbiome in selected rainbow trout could provide novel insights into the adaptation mechanisms of the fish to all-plant protein feeds. Nowadays, it is widely accepted that there is a correlation between fish genotype and gut microbial communities, but to date, there is very little information on how genetic selection drives differences in intestinal microbiota composition and how this could affect diet plasticity, health, and disease resistance in both marine and freshwater fish species (Blaufuss et al., 2020; Piazzon et al., 2020).

Therefore, the present study was designed to investigate the growth performance, whole-body proximate composition, and intestinal microbiome of rainbow trout strains when selected and non-selected for weight gain on all-plant protein feeds in response to all-plant protein and commercial low-FM diets.

## Materials and Methods

### Experimental Fish and Diets

A feeding trial was conducted at the Experimental Facility of the Department of Agricultural, Forest and Food Sciences (DISAFA) of the University of Turin (Italy) using two different rainbow trout strains. The selected strain (United States) was developed by the United States Department of Agricultural–Agricultural Research Service (USDA-ARS) and the University of Idaho by introgression of nine domesticated commercial and conservation stocks (Overturf et al., 2013). The selection was based on growth performance when trout were fed an all-plant protein feed. Besides dietary utilization, the selected strain also displays an enhanced non-specific pathogen resistance and a concomitant resistance to the development of distal intestine enteritis (Venold et al., 2012; Abernathy et al., 2017). The non-selected strain (ITA) was selected for different performance traits when fed a commercial low-FM diet by the Italian hatchery Fratelli Leonardi (Trento, Italy). To understand the effects of both diet and genotype, a 2x2 factorial design was applied. In particular, each rainbow trout strain was fed using two different experimental diets: a diet containing only plant proteins (PP, provided by the University of Idaho), and a commercial low-FM diet (C) containing both the plant meals and FM (provided by Naturalleva, Cologna Veneta, Verona, Italy). No information was provided by the feed company about the C diet formulation as it was considered confidential. Both the diets were provided as extruded feeds.

In detail, the United States embryonated eggs were received from the University of Idaho and, after hatching in hatchery tanks, were fed commercial diets (crumbled extruded feeds of different sizes, with 55–62% crude protein [CP] and 11–18% ether extract [EE]; Skretting Italia Spa, Mozzecane, Verona, Italy) until the beginning of the experimental trial. In parallel, ITA fingerlings (mean weight of 2.0 ± 0.01 g) were provided by Fratelli Leonardi and fed the same commercial diets until the beginning of the feeding trial as well. After reaching the juvenile stage, 250 juvenile rainbow trout (125 from each strain) underwent light anesthesia (MS-222; PHARMAQ Ltd., United Kingdom; 60 mg/L) and were individually weighed (10.63 ± 0.01 g) by using electronic scales (KERN PLE-N v. 2.2; KERN and, Sohn GmbH, Balingen-Frommern, Germany; d: 0.1). Then, fish from each strain were randomly distributed into ten 100-L, rectangular-shaped tanks (five replicate tanks per strain, 25 fish per tank) connected to a flow-through open system supplied with artesian well water (constant temperature of 13 ± 1°C, 8 L min-1, dissolved oxygen ranged between 7.6 and 8.7 mg L), and fed the C diet (USA-C and ITA-C treatments). In parallel, at the same rearing conditions, 200 juvenile rainbow trout (100 from each strain; initial body weight of 13.47 ± 0.01 g) were randomly allotted to ten 100-L, rectangular-shaped tanks (five replicate tanks per strain, 20 fish per tank), and fed the PP diet (USA-PP and ITA-PP treatments). In order to update the daily amount of feed, the biomass in each tank was weighed in bulk every 14 days. Fish were fed by hand twice a day (08:00 and 15:00) and 6 days per week, up to a maximum of 2.5% of the tank biomass the first 6 weeks of the feeding trial. Then, according to the fish growth, the daily amount of feed per tank was progressively reduced to 1.7%. Feed intake was checked at each administration, as feed distribution was immediately suspended when fish stopped eating. Undistributed feed was weighed and this data was used for the correct calculation of the growth parameters. Mortality was daily recorded. The feeding trial lasted 105 days.

### Chemical Analyses of Feed

Feed samples were ground using a cutting mill (MLI 204; Bühler AG, Uzwil, Switzerland) and analyzed for dry matter (DM, AOAC #934.01), CP (AOAC #984.13), and ash (AOAC #942.05) contents according to AOAC International (2000). Feed samples were also analyzed for EE (AOAC #2003.05) content according to AOAC International (2003). The gross energy (GE) content was determined using an adiabatic calorimetric bomb (C7000; IKA, Staufen, Germany). All the chemical analyses of the feeds were performed in duplicate. The proximate composition of the experimental diets is shown in Table 1.

**TABLE 1:** Feed ingredients (g/kg, as fed) and proximate composition of the experimental diets.

	PP	C
Ingredients (g/kg, as fed)		
Soy protein concentrate	265.8	-
Soybean meal, dehulled	250.0	-
Corn gluten meal (CP 60%)	170.0	-
Wheat gluten meal	29.2	-
Fish oil	194.2	-
L-lisin HCl	18.4	-
DL-methionine	4.4	-
Threonine	2.0	-
Taurine	5.0	-
Vitamin premix^a^	10.0	-
Stay C 35%	2.0	-
Dicalcium phosphate	15	-
Potassium chloride	5.6	-
Sodium chloride	2.8	-
Monocalcium phosphate	24.6	-
Trace mineral premix^b^	1.0	-
Proximate composition^c^		
DM, g/kg	91.32	90.71
CP, g/kg DM	48.97	49.71
EE, g/kg DM	18.11	26.56
Ash, g/kg DM	6.04	7.19
GE, MJ/Kg^d^	22.80	24.07

DM, dry matter; CP, crude protein; EE, ether extract; GE, gross energy; ^a^Vitamin premix supplied the following per kg diet: vitamin A, 2.4 mg; vitamin D, 0.15 mg; vitamin E, 267 mg; vitamin K as menadione sodium bisulfite, 20 µg; thiamin as thiamin mononitrate, 32 mg; riboflavin, 64 mg; pyridoxine as pyridoxine-HCl, 64 mg; pantothenic acid as Ca-d-pantothenate, 192 mg; niacin as nicotinic acid, 240 mg; biotin, 0.56 mg; folic acid, 12 mg; vitamin B_12_, 50 µg; and inositol as meso-inositol, 400 mg ^d^Trace mineral premix supplied the following (mg/kg diet): Zn (as ZnSO_4_.7H_2_O), 75; Mn (as MnSO_4_), 20; Cu (as CuSO_4_.5H_2_O), 1.54; I (as KIO_3_), 10. ^c^Values are reported as mean of duplicate analyses; ^d^Determined by calorimetric bomb.

### Growth Performance

At the end of the feeding trial, the feed was withheld from the fish for 1 day, and then they were individually weighted after light anesthesia. The following performance indexes were calculated per tank:

Survival (%) = 100—[(number of dead fish/number of fish at start) × 100].

Individual weight gain (iWG, g) = individual mean final body weight (iFBW, g)—individual mean initial body weight (iIBW, g).

Feed conversion ratio (FCR) = total feed supplied (g, DM)/WG (g).

Protein efficiency ratio (PER) = WG (g)/total protein fed (g, DM).

Specific growth rate (SGR, % day-1) = [(lnFBW–lnIBW)/number of days] × 100.

### Whole-Body Proximate Composition

At the end of the feeding trial, 3 fish per tank (15 fish per dietary treatment) were sacrificed by an overdose of anesthetic (MS-222, PHARMAQ Ltd., United Kingdom; 500 mg/L) and then frozen at -20°C. Frozen fish were finely ground with a knife mill (Grindomix 174 GM200; Retsch GmbH, Haan, Germany) to obtain one pool of three fish per tank (5 pools per dietary treatment). All the fish pools were freeze-dried and used for the determination of the final whole-body proximate composition. The proximate composition of fish whole body was determined following the same procedures used for feed analyses (AOAC International, 2000 and 2003).

### Sampling and Processing

At the end of the feeding trial, six fish per dietary treatment were sacrificed by an overdose of anesthetic, and the whole intestine was aseptically dissected out. The gut autochthonous microbiota was obtained by scraping the mucosa of the entire intestine (excluding pyloric caeca) with a sterile swab as reported in detail by Rimoldi et al. (2019). Briefly, each swab head was cut and immediately transferred to a sterile 1.5 ml microtube containing 200 μl of Xpedition Lysis/Stabilization Solution. The tube was then vortexed for shaking out the bacteria from the swab tip and stored at room temperature for up to 24 h until bacterial DNA extraction.

### Bacterial DNA Extraction

The DNA was extracted from 200 µl of bacterial suspension and from three aliquots (200 mg) of each experimental feed using the DNeasy PowerSoil® Kit (Qiagen, Milan, Italy), according to the manufacturer’s instructions. The samples were mechanically lysed in PowerBead Tubes by means of a TissueLyser II (Qiagen) for 2 min at 25 Hz. The extracted DNA was spectrophotometrically quantified using the NanoDropTM 2000 spectrophotometer (Thermo Scientific, Milan, Italy) and stored at -20°C until the PCR reaction was performed.

### 16S rRNA Gene Amplicon Library Preparation and Sequencing

Preparation of the 16S metagenomic library and sequencing were performed by GalSeq srl (Italy), targeting the variable V4 region and applying Illumina protocol “16S Metagenomic Sequencing Library Preparation for Illumina MiSeq System” (#15044223 rev. B). Details of the methodology used have previously been described (Rimoldi et al., 2019; Terova et al., 2019). Briefly, primers are tailed with sequences to incorporate indexing barcodes, and samples are pooled into a single library for sequencing on the Illumina MiSeq device using the pair-ended sequencing (2 × 250) strategy. All FastQ sequence files were submitted to the European Nucleotide Archive (EBI ENA).

### Analysis of Metabarcoding Data

The raw sequence files were imported in QIIME™ 2 (v. 2018.4) (Bolyen et al., 2019) and processed at the default setting. Artefacts and primers were trimmed, the remaining sequences were filtered for base quality (Q > 30), and forward and reverse reads were merged. Filtered reads were then dereplicated and chimeras removed using the denoise-paired command of the DADA2 plug-in. The output was an amplicon sequence variant (ASV) table, analog to the OTU table, which records the number of times each exact ASV was observed in each sample. The representative sequences were classified using the Silva reference database (https://www.arb-silva.de/) down to the genus level. All sequences assigned to eukaryotes (chloroplasts and mitochondria) in gut samples were discarded. Alpha and beta diversity analyses were performed using QIIME scripts “alpha_rarefection.py” and “jackknifed_beta_diversity_.py”, respectively. Principal Coordinates Analysis (PCoA) based on both unweighted UniFrac and weighted UniFrac distance matrices (Lozupone and Knight, 2005; Lozupone et al., 2007) was conducted to visualize similarities or dissimilarities between bacterial communities.

### Predictive Functional Analysis of Bacterial Communities

The PICRUSt (Phylogenetic Investigation of Communities by Reconstruction of Unobserved States) software package was used to predict the functional profiling of microbial communities on the basis of 16S rRNA gene sequences (Langille et al., 2013) as previously described (Rimoldi et al., 2021). The inferred metagenomic functions were predicted using the Kyoto Encyclopedia of Genes and Genomes (KEGG database). The maximum allowed Nearest Sequenced Taxon Index (NSTI) value was set to two. Analysis of the output generated by PICRUSt was made using the Statistical Analysis of Metagenomic Profiles (STAMP) software package (Parks et al., 2014) applying a two-sided Welch t-test (*p* < 0.05) to identify differences in microbial metabolic pathways between two groups.

### Bioinformatics and Statistical Analysis

The experimental unit for statistical analyses was the tank for growth performance and whole-body proximate composition, and the fish for microbiome analysis.

The statistical analysis of growth performance and whole-body proximate composition was performed using IBM SPSS Statistics v. 28.0 (IBM, Armonk, NY, United States). The Shapiro–Wilk test assessed the normality distribution of the residuals, while the assumption of equal variances was assessed by Levene’s homogeneity of variance test. The growth performance and whole-body proximate composition data were analyzed by fitting a general linear model that allowed them to depend on two fixed factors (strain and diet) and their interaction. The interactions between the levels of the fixed factors were evaluated by pairwise contrasts. The results obtained were expressed as the least square mean and pooled standard error of the mean (SEM). *p* values ≤ 0.05 were considered statistically significant.

The statistical analysis of gut microbiome data was performed using Past3 software (Hammer et al., 2001). All the data were checked for normality and homogeneity of variance using Shapiro–Wilk and Levene’s test, respectively. Concerning gut microbiota composition, only those taxa with an overall abundance of more than 0.5% (up to order level) and 0.01% at family and genus level were considered for statistical analysis. Before being statistically analyzed, the percentage values were angular transformed (arcsine of the square root). To test the null hypothesis (*p* < 0.05), a two-way ANOVA was performed considering the influence of two main factors (strain and diet) and their interaction. Multivariate analysis of beta diversity was tested using a two-way non-parametric permutational multivariate analysis of variance (two-way PERMANOVA) with 999 permutations.

## Results

### Growth Performance

The growth performance of the rainbow trout in the present study is summarized in Table 2. Survival was high for all the dietary treatments (range: 95.00–100), with a significant influence of both the strain (*p* < 0.05) and the interaction between strain and diet (*p* < 0.001) being identified. In particular, the United States rainbow trout showed higher survival than the ITA group (*p* < 0.05), but greater survival was observed in the United States strain fed the PP diet when compared to the PP-fed ITA fish only (100.00 ± 0.51 vs. 95.00 ± 0.51; *p* < 0.001). In parallel, the ITA rainbow trout fed the C diet displayed higher survival than the C-fed United States group (99.20 ± 0.51 vs. 96.80 ± 0.51; *p* = 0.001). Higher survival was also observed in both the United States and the ITA strains when fed the PP and C diets, respectively (*p* < 0.001). Differently, the iFBW was influenced by both strain and diet (*p* < 0.001). The United States fish showed greater iFBW than the ITA group (*p* < 0.001), with higher values being also observed when the fish were fed the PP diet in comparison with the C one (*p* < 0.001). The iWG depended on all the considered variables (strain and diet: *p* < 0.001; strain × diet: *p* = 0.010). In particular, the United States rainbow trout showed higher iWG than the ITA group (*p* < 0.001), with greater iWG being also observed when fish were fed the PP diet in comparison with the C one (*p* < 0.001). However, the United States rainbow trout displayed higher iWG than the ITA strain when they were fed the C diet only (55.12 ± 1.25 vs. 47.43 ± 1.25; *p* < 0.001). The FCR and the PER were not affected by any of the considered variables (*p* > 0.05). Finally, the SGR depended on strain only (*p* < 0.001), with higher values being identified in the United States rainbow trout in comparison with the ITA fish (*p* < 0.001).

**TABLE 2:** Growth performance of the rainbow trout depending on strain, diet, and their interaction (*n* = 5).

	Strain (S)		Diet (D)		SEM		*p* value		
	ITA	United States	C	PP	S	D	S	D	S × D
Survival, %	97.10	98.40	98.00	97.50	0.36	0.36	0.010	0.323	<0.001
iFBW, g	69.82	76.18	61.18	84.82	0.80	0.80	<0.001	<0.001	0.628
iWG, g	54.96	59.40	51.27	63.09	0.88	0.88	<0.001	<0.001	0.010
FCR, n	0.96	0.94	0.96	0.94	0.02	0.02	0.176	0.345	0.171
PER, n	2.04	2.10	2.10	2.04	0.03	0.03	0.173	0.240	0.162
SGR, % day-1	1.61	1.70	1.66	1.65	0.01	0.01	<0.001	0.634	0.371

ITA, Italian strain; United States, American strain; C, commercial diet; PP, all-plant protein diet; SEM, standard error of the mean; P, probability; iIBW, individual initial body weight; iFBW, individual final body weight; iWG, individual weight gain; SGR, specific growth rate; FCR, feed conversion ratio; PER, protein efficiency ratio.

### Whole-Body Proximate Composition

Table 3 summarizes the whole-body proximate composition of the rainbow trout in the current research. The moisture depended on all the considered variables (strain and diet: *p* < 0.001; strain × diet: *p* = 0.042). In particular, the ITA rainbow trout showed lower moisture content than the United States group (*p* < 0.001), with a reduction in the whole-body moisture being also observed when fish were fed the PP diet in comparison with the C one (*p* < 0.001). However, the ITA strain displayed lower moisture content than the United States rainbow trout when they were fed the PP diet only (70.83 ± 0.25 vs. 72.43 ± 0.25; *p* < 0.001). The ash content was influenced by the strain × diet interaction only (*p* < 0.05), with the C-fed United States fish displaying increased whole-body ash in comparison with the USA-PP group (2.52 ± 0.07 vs. 2.26 ± 0.07; *p* < 0.05). Differently, the CP content uniquely depended on diet (*p* < 0.001). In detail, the PP diet led to higher whole-body CP than the C feed (*p* < 0.001). On the contrary, the EE content was affected by all the considered variables (*p* < 0.001). In particular, the ITA fish displayed higher whole-body EE than the United States strain (*p* < 0.001), with an increase in the EE content being also observed when fish were fed the PP diet in comparison with the C one (*p* < 0.001). However, greater whole-body EE was observed in the ITA rainbow trout fed the PP diet when compared to the PP-fed United States group only (9.84 ± 0.10 vs. 8.28 ± 0.10; *p* < 0.001). Furthermore, the ITA strain showed increased EE content when fed the PP diet (9.84 ± 0.10 vs. 8.71 ± 0.10 [C]; *p* < 0.001).

**TABLE 3:** Final whole-body proximate composition (as is) of the rainbow trout depending on strain, diet, and their interaction (*n* = 5).

	Strain (S)		Diet (D)		SEM		*p* value		
	ITA	United States	C	PP	S	D	S	D	S × D
Moisture, %	71.71	72.82	72.90	71.63	0.17	0.17	<0.001	<0.001	0.042
Ash, %	2.42	2.39	2.46	2.35	0.05	0.05	0.622	0.139	0.031
CP, %	16.25	16.16	15.75	16.66	0.16	0.16	0.652	<0.001	0.652
EE, %	9.27	8.38	8.60	9.06	0.07	0.07	<0.001	<0.001	<0.001

ITA, Italian strain; United States , American strain; C, commercial diet; PP1, all-plant protein diet; SEM, standard error of the mean; P, probability; DM, dry matter; CP, crude protein; EE, ether extract.

### Microbial DNA Sequencing

Illumina sequencing of the 30 samples (24 mucosae + six feeds) yielded 1,015,591 high-quality reads, which were successfully taxonomically assigned according to the Silva database. The average number of reads (mean + SD) per sample was 24,838 ± 5,381 and 37,356 ± 4,810 for feed and gut mucosa samples, respectively (Supplementary data file S1). Rarefaction analysis of the chao1 index showed most of the curves approximating the saturation, with one intestinal sample from group United States_C failing only (Supplementary Figure S1). To ensure the adequateness of the remaining samples, the good coverage was also calculated, resulting in a good range (ranged 0.99–1) in all the samples.

When comparing the bacterial alpha diversity of gut mucosa samples, no significant differences were found in species richness and biodiversity indices (Table 4). Differently, the plant protein feed-related microbiota was characterized by higher species richness (Chao1 and Faith_PD) and lower diversity (Shannon and Simpson indices) than the C diet (Table 4).

**TABLE 4:** Alpha diversity indices (mean ± SD, rarefied at 17,778 reads) of microbial communities of feed (*n* = 3) and gut mucosa (*n* = 6) samples. diet, D; strain, S.

	Chao 1	Faith_PD	Observed OTUs	Shannon	Simpson
ITA_C	81 ± 46	1.41 ± 0.58	74 ± 43	3.69 ± 0.26	0.85 ± 0.02
ITA_PP	73 ± 36	1.27 ± 0.24	66 ± 33	3.61 ± 0.14	0.84 ± 0.01
USA_C	97 ± 45	1.27 ± 0.23	86 ± 39	3.83 ± 0.43	0.85 ± 0.03
USA_PP	118 ± 34	1.40 ± 0.38	104 ± 35	3.88 ± 0.52	0.85 ± 0.03
*Sig*	D: 0.661	D: 0.965	D: 0.737	D: 0.961	SxD: 0.676
	S: 0.081	S: 0.985	S: 0.123	S: 0.184	S: 0.431
	SxD: 0.401	SxD: 0.411	SxD: 0.423	SxD: 0.676	SxD: 0.739
Feed_C	269 ± 7	3.47 ± 0.11	260 ± 7	4.92 ± 0.02	0.90 ± 0.00
Feed_PP	288 ± 1	4.05 ± 0.22	261 ± 7	4.66 ± 0.12	0.89 ± 0.01
*t-test*	0.010	0.014	0.827	0.024	0.010

ITA, Italian strain; United States , American strain; C, commercial diet; PP, all-plant protein diet.

### Feed-Related Microbial Community Profiles

**FIGURE 1:**
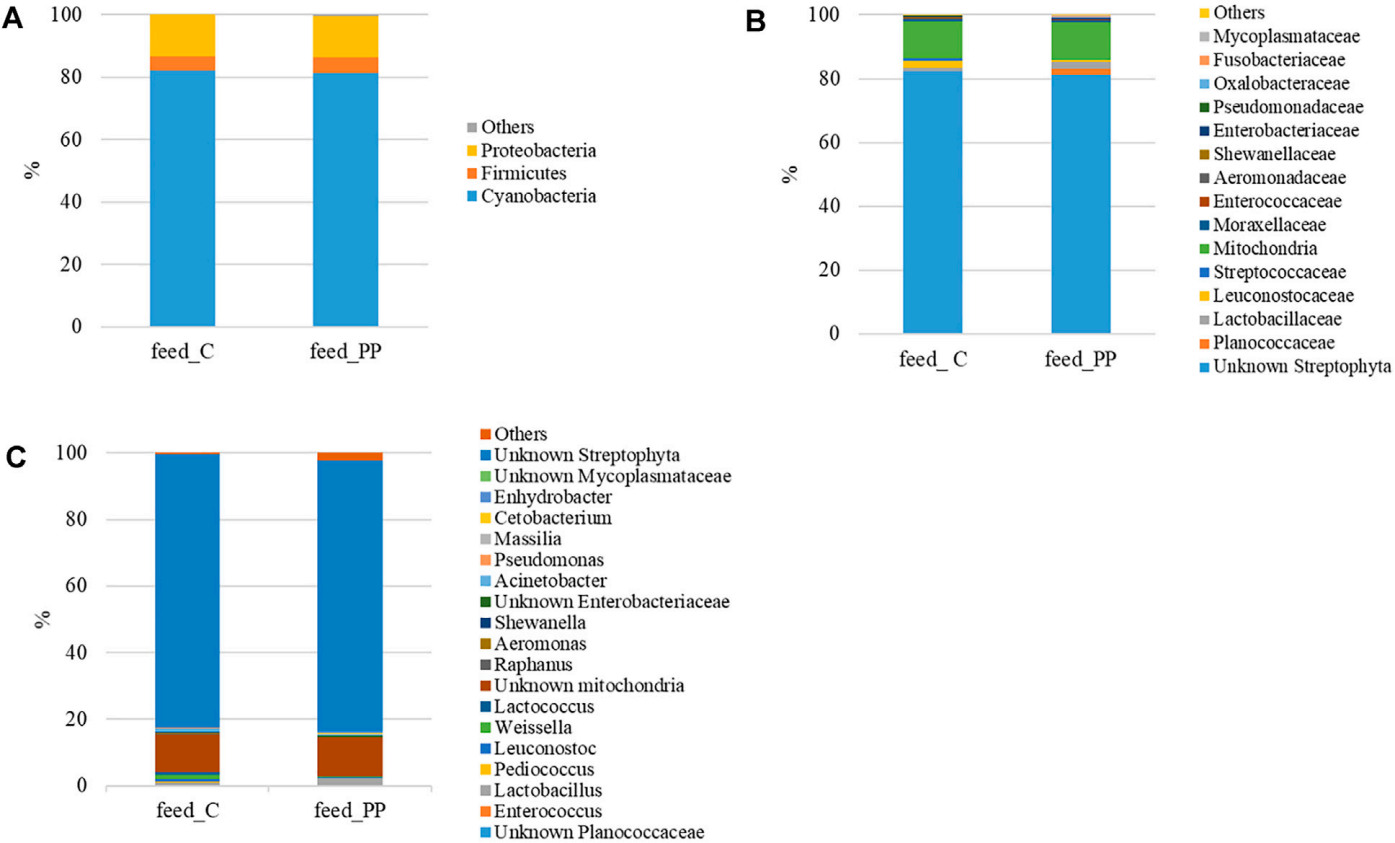
Mean relative abundance (%) of the most prevalent bacterial taxa in experimental feeds at (*n* = 3) phylum **(A)**, family **(B)**, and genus **(C)** levels.

The microbial community profiles of the feeds were outlined up to the genus level. Most of the sequences (81–82%) were of plant origin (p_Cyanobacteria and c_Chloroplast), deriving from vegetable ingredients of the diets. Considering only the most representative taxa, the overall feed-related microbial community comprised three phyla, four classes, five orders, 14 families, and 14 genera. The profiles of the feed-related microbial communities are shown at the phylum, family, and genus level in Figure 1. The list of the most abundant bacterial taxa, their relative abundance values, and statistical analysis are reported in Supplementary Table S1. Proteobacteria and Firmicutes were the dominant bacterial phyla in feeds, with a relative abundance of 13% and 4%, respectively (Figure 1A).

Accordingly, Alphaproteobacteria and Bacilli were the predominant classes of bacteria in the feed samples. At the order level, Lactobacillales only differed between the two feeds, resulting in two times more abundant in the C diet than in the PP feed. Bacteria assigned to the Leuconostocaceae, Streptococcaceae, Aeromonadaceae, and Shewanellaceae families were preferentially associated with the C diet, while Fusobacteriaceae and Mycoplamataceae were found in the PP feed only (Figure 1B). Similarly, at the genus level, *Pediococcus*, *Leuconostoc*, *Weissella*, *Lactococcus*, *Aeromonas*, *Shewanella*, and *Acinetobacter* were significantly higher in the C diet when compared to the PP feed (Figure 1C). The relative abundance of the *Lactobacillus* genus only was increased in the PP diet, whereas *Cetobacterium* and *Anhydrobacter* genera were solely detected (Figure 1C).

### Effects of Genotype and Diet on the Gut Microbiome Composition

**FIGURE 2:**
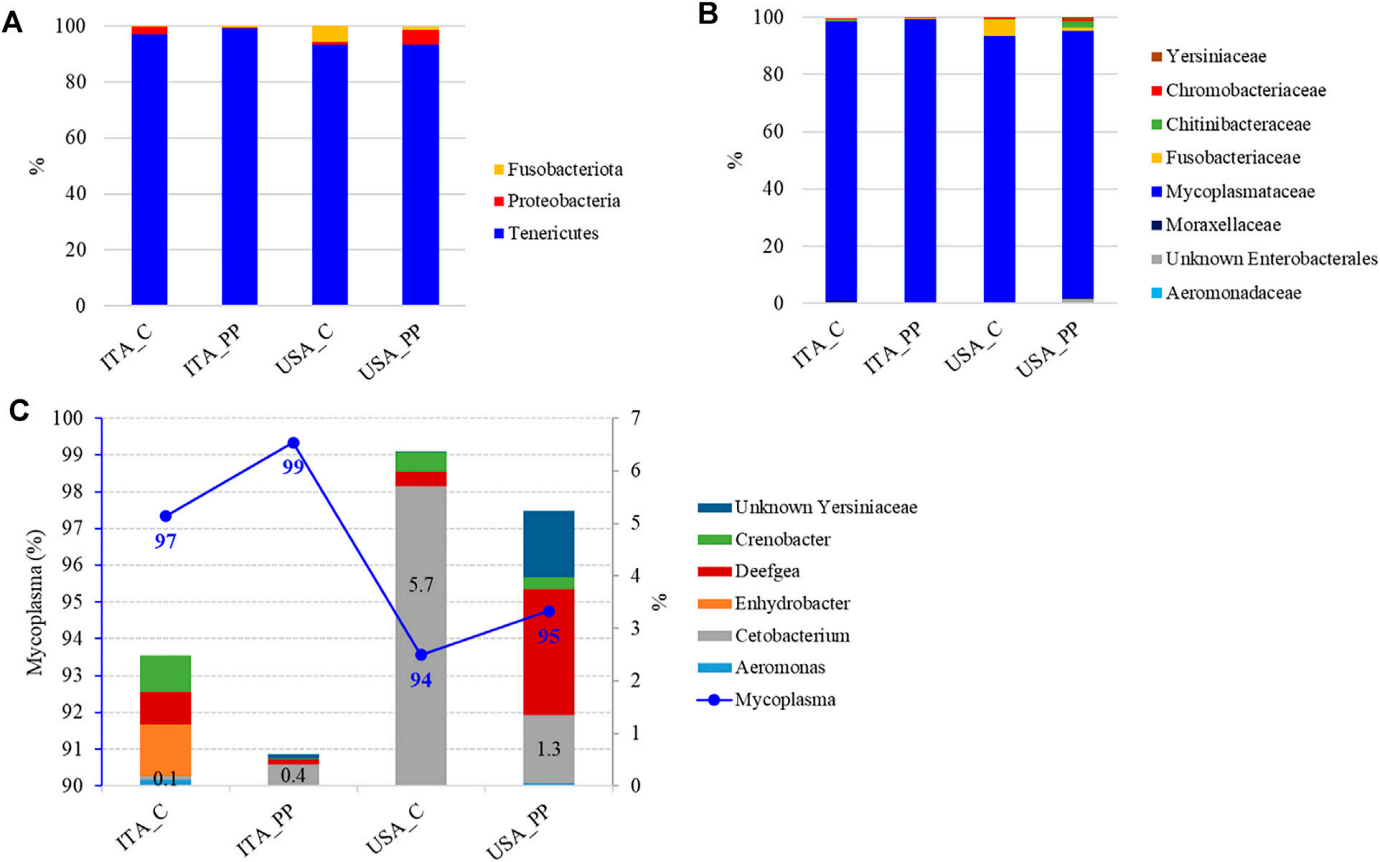
Mean relative abundance (%) of the most prevalent bacterial taxa in gut mucosa of two rainbow trout strains (the United States and ITA) fed experimental diets (C and PP) at phylum **(A)**, and family **(B)**, and genus **(C)** level. ITA_C (*n* = 6), ITA_PP (*n* = 6), United States_C (*n* = 5), and United States_PP (*n* = 6). Percentages of the Cetobacterium genus are indicated on histograms. Blueline and the corresponding values indicate the amount of Mycoplasma in each experimental feeding group.

The overall intestinal microbial community, considering only the most representative taxa, was mainly composed of three phyla, three classes, five orders, seven families, and six genera. The profiles of the intestinal microbial communities for each dietary treatment are shown at phylum, family, and genus level in Figure 2. The most abundant bacterial taxa with their relative abundance data and statistical analysis are listed in Supplementary Table S2.

**FIGURE 3:**
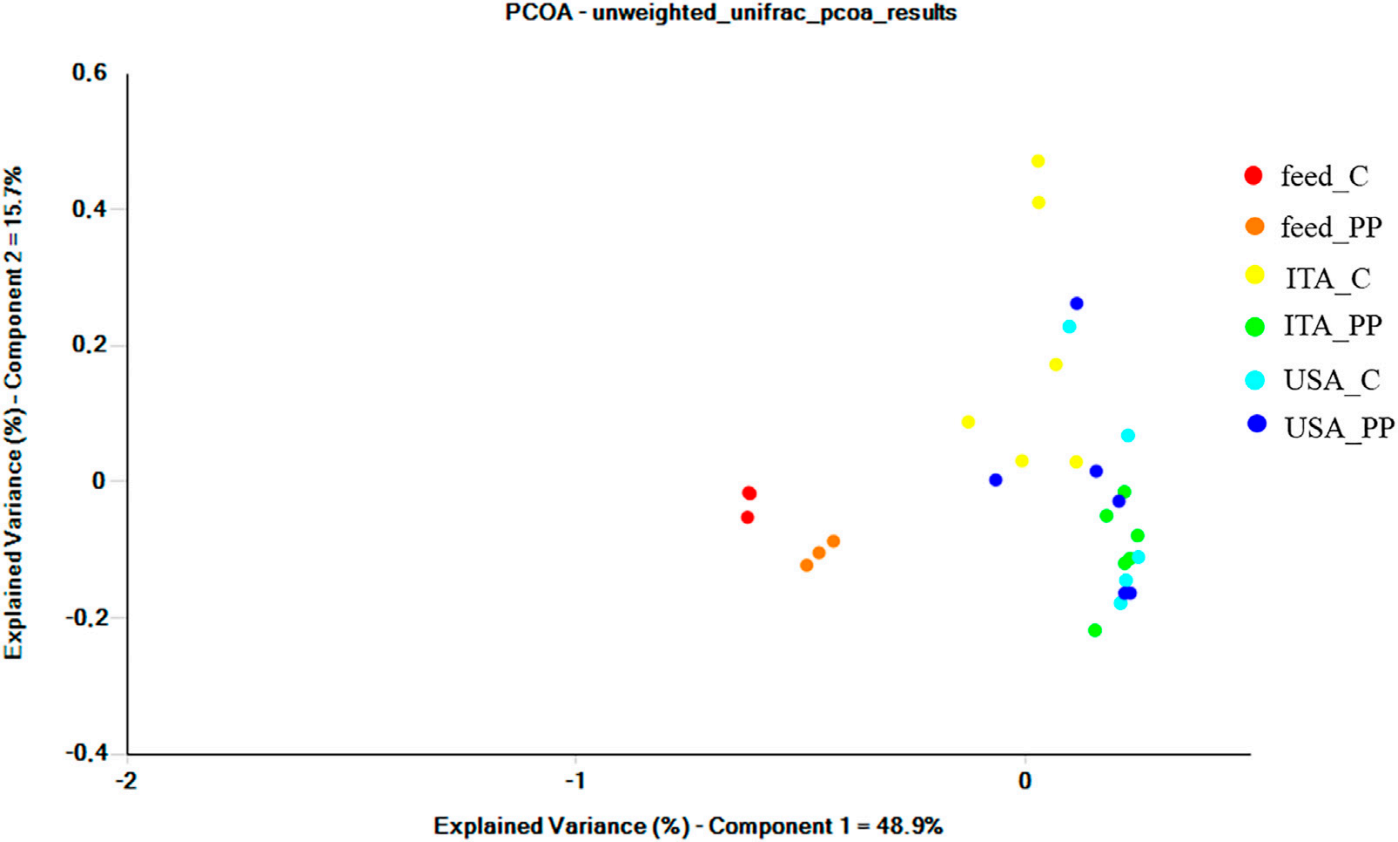
Beta diversity metrics. Principal coordinate analysis (PCoA) of unweighted UniFrac distances of microbial communities associated with gut mucosa and feeds. The figure shows the bi-dimensional plot of individual fish and feeds samples according to their microbiome profile.

To determine the impact of strain and diet on intestinal phylotypic diversity, UniFrac analysis was performed. Principal Coordinates Analysis (PCoA) of unweighted UniFrac distances revealed an effect of both the strain and the diet on gut microbial community profiles, with the first principal coordinate PC1 explaining up to 48.9% of the variation among the individuals (Figure 3).

Intestinal samples appeared clearly separated from the feed ones, thus indicating that observed differences between intestinal bacterial communities were not simply a consequence of undigested feed-related bacteria. Gut communities from the ITA strain clustered separately according to the diet. Similarly, distinct clusters were observed between the trout strains fed the C diet. Results of unweighted UniFrac PCoA were wholly confirmed by the non-parametric multivariate statistical test PERMANOVA (Table 5). The unweighted data showed a significant interaction between strain and diet. Results of pairwise comparisons showed differences between strains fed the same diet, while an overall effect of the diet was evident for the ITA strain only (*p* = 0.004). The weighted UniFrac analysis was also performed, but no significant differences were found among the groups (data not shown).

**TABLE 5:** Results of non-parametric multivariate analysis PERMANOVA on the unweighted UniFrac data of intestinal and feed samples. Significant *p* values (*p* ≤ 0.05) are in bold.

Two-way PERMANOVA		
Permutation N	999	
Source	Pseudo-F	P
Strain	1.354	0.220
Diet	4.800	**0.021**
Interaction	3.904	**0.012**
Pairwise comparisons		
	Pseudo-F	P
Feed C vs. feed PP	48.5	0.103
ITA_C vs. ITA_PP	5.435	**0.004**
ITA_C vs. USA_C	3.296	**0.022**
ITA_C vs. USA_PP	1.964	0.101
ITA_PP vs. USA_C	1.933	0.095
ITA_PP vs. USA_PP	2.339	**0.014**
USA_C vs. USA_PP	1.245	0.264

ITA, Italian strain; United States , American strain; C, commercial diet; PP, all-plant protein diet; P, probability.

The autochthonous gut microbiome of rainbow trout was mostly dominated, regardless of the diet, by the Tenericutes phylum, mainly represented by the *Mycoplasma* genus, with a relative abundance ranging between 93 and 99% in all the samples (Figure 2). Proteobacteria and Fusobacteria completed the microbiota profile, while Firmicutes were unexpectedly not detected. The difference in gut microbiota composition between the two strains was limited to the *Cetobacterium* genus, belonging to the Fusobaceriaceae family (Figure 2). Two-way ANOVA indicated that the relative abundance of this genus was influenced by genotype only, with results being higher in the United States strain when compared to the ITA fish (Figure 2, Supplementary Table S2).

### Functional Analysis of the Gut Microbiome

**FIGURE 4:**
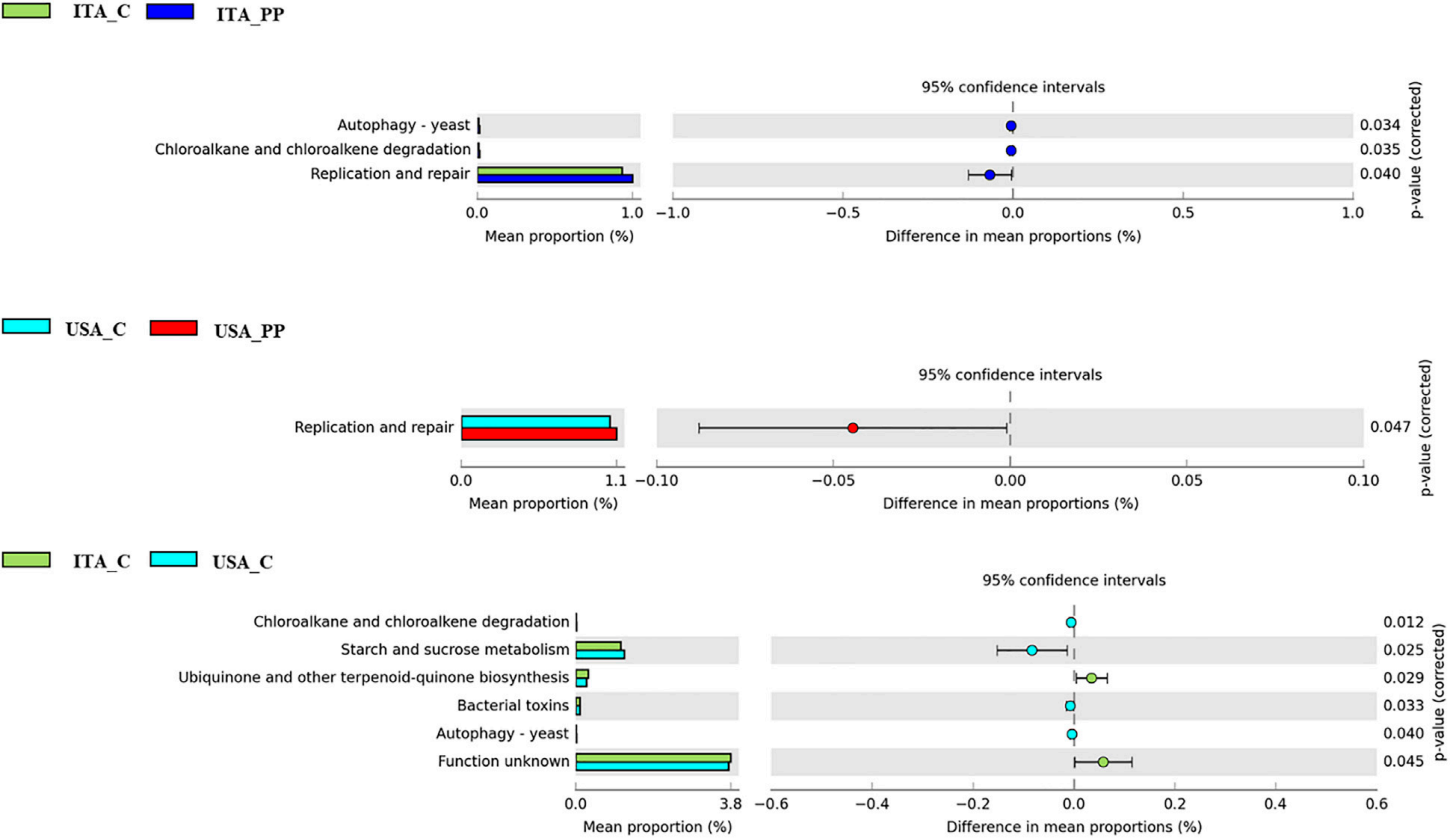
Extended error bar plots showing PICRUSt-predicted bacterial KEGG pathways statistically different between experimental groups.

To attain a comprehensive analysis of the functional composition of the metagenome of each gut microbial community based on 16S rRNA sequencing, the PICRUSt method was used at Level 3 KEGG. Comparative analysis of PICRUSt functional inferences revealed an increased abundance of genes responsible for replication and repair pathway in both the ITA and the United States strains fed the all-plant protein diet (Figure 4). A total of six pathways were, instead, significantly different between the two strains when fed the C feed. Specifically, the pathway involved in starch and sucrose metabolism was more abundant in the United States trout in comparison with the ITA strain. On the contrary, the ITA trout showed an over-representation of the ubiquinone biosynthesis pathway (Figure 4).

## Discussion

### Growth Performance

Independent of the administered diet, the United States rainbow trout strain in the present study overall showed better growth performance (in terms of increased FBW, iWGm, and SGR) when compared to the ITA one. Furthermore, a clear strain×diet interaction indicating an improvement in the iWG in the United States fish was observed when the C diet only was provided. This represents an interesting finding, as the selected strains usually outperform the non-selected ones on the plant-based selection diet (Overturf et al., 2013). Therefore, the ability of the United States rainbow trout to grow more efficiently when fed either the low-FM or the all-plant protein diets–with even more pronounced effects when using the former–reflects the genetic selection directed towards the utilization of the vegetable proteins, which efficiently integrates the physiological, carnivorous feeding behavior of this fish species. Analogously to what was reported by Overturf et al. (2013), the selected strain of the current research displayed similar FCR to that of the non-selected one, thus still not excluding a primary role of the feed intake–rather than the feed efficiency–in the improvement of the growth performance (Callet et al., 2017). Differently, the percent survival of the rainbow trout of the present study–while remaining high (≥ 95%)—displayed a clear strain × diet interaction, with the United States strain surviving more when fed the all-plant protein diet, and the ITA fish behaving similarly when fed the low-FM diet. This is in partial agreement with Callet et al. (2017) that observed higher survival in the selected strain fed the plant-based diets in comparison with the non-selected fish, thus reasonably reflecting genetic selection. As a final aspect to consider, independently of the strain, the rainbow trout used in the current research showed greater FBW and iWG when fed the PP diet. This may be related to the high protein digestibility of all-plant protein diets that have previously been highlighted, as such diets are usually formulated with highly digestible protein concentrates (Gaylord et al., 2008; Callet et al., 2017; Brezas and Hardy, 2020). Furthermore, the ability of rainbow trout to grow efficiently on a diet containing soy protein concentrate-based blend as a replacement of high percentages (until 87%) of the FM in the formulation has already been reported (Burr et al., 2012).

### Whole-Body Proximate Composition

Independent of the considered strain, the PP diet led to the lower whole-body moisture content in comparison with the C one, as previously reported (Overturf et al., 2013). Furthermore, a clear strain × diet interaction indicating a reduction in the moisture content of the ITA fish was observed when the PP diet only was provided. This may represent the logical consequence of the increased whole-body EE identified in the PP-fed ITA rainbow trout when compared to the United States fish fed the same diet. As far as ash content is concerned, the C diet determined an overall increase in the United States strain only. Overturf et al. (2013) previously reported that whole-body ash was unaffected in either the selected or the non-selected rainbow trout in response to both a FM- or PP-based diet, but no information about selection-related ash digestibility changes is currently available. Therefore, the increased ash content herein highlighted may result from a better ash digestibility, thus potentially strengthening the effect of the higher ash content of the C diet than the PP feed. Differently, the United States strain showed that the whole-body CP content was similar to the non-selected one, as previously reported (Overturf et al., 2013). This also confirms that CP digestibility is not different in the selected rainbow trout as previously observed (Callet et al., 2017; Brezas and Hardy, 2020). However, independently of the considered strain, the PP diet led to an increased CP content in comparison with the C feed, thus reflecting the above-mentioned high digestibility of the vegetable proteins (Gaylord et al., 2008; Callet et al., 2017; Brezas and Hardy, 2020). As a final aspect to consider, the whole-body EE content of the rainbow trout of the present study displayed a clear strain×diet interaction, with the ITA strain body containing more EE than the United States fish when fed the all-plant protein diet. Genetic selection towards the utilization of vegetable proteins has been reported to not influence the whole-body EE content of rainbow trout (Overturf et al., 2013; Callet et al., 2017; Brezas and Hardy, 2020). However, the higher whole-body EE content of the PP-fed ITA rainbow trout when compared to the United States fish may result from a synergic effect of both the physiological response to all-plant protein diets (Burel et al., 1998; Kaushik et al., 2004; Lazzarotto et al., 2015) and the use of a C diet (characterized by higher EE content) across the generations.

### Microbiome Analysis

The high-throughput sequencing on the Illumina MiSeq platform has been used in the present research for intestinal microbiome characterization. In line with the previous studies performed on rainbow trout, results of the metabarcoding analysis showed that the most abundant phylum of autochthonous gut microbiota in rainbow trout, regardless of the diet and genotype, was Tenericutes, mainly represented by the *Mycoplasma* genus (Rimoldi et al., 2019). Accordingly, it has recently been suggested that *Mycoplasma*, due to its dominance in the distal intestine of rainbow trout and other farmed salmonids, could have a mutualistic relationship with its host (Rasmussen et al., 2021). In Atlantic salmon (*Salmo salar*), the abundance of *Mycoplasma* has positively been associated with improved growth, carotenoid utilization, and disease resilience of the host (Bozzi et al., 2021). In addition, this genus harbors genes for long-chain polymer degradation, such as chitin, which is particularly abundant in insects, that are part of the natural diet of juvenile salmonids. This ability of *Mycoplasma* could be beneficial for its host, which can utilize all the nutritional value of a chitin-rich diet. Indeed, an increased presence of *Mycoplasma* was also shown in the gut of rainbow trout fed an insect-based diet (Rimoldi et al., 2019). An unexpected result was the absence of bacteria belonging to the Firmicutes phylum in all the gut samples, even in those fish fed the plant-based diet. Members of this phylum are known to play a key role in the fermentation of dietary carbohydrates (Corrigan et al., 2015), and are usually associated with plant ingredients, while low-FM diet generally favors the presence of Proteobacteria (Desai et al., 2012; Ingerslev et al., 2014a and b; Rimoldi et al., 2018). An increase in Firmicutes to Proteobacteria ratio was, however, observed by Blaufuss et al. (2020) in analogously selected trout for growth on an all-plant protein diet when fed all-plant protein feeds.

In terms of alpha diversity, no differences were detected between ITA and United States strains in microbial richness and biodiversity, regardless of the administered diet. Comparison of the overall microbial composition among all the samples according to weighted and unweighted UniFrac showed an overlap among all the gut samples, with the feed samples clustering separately. The composition of feed-related microbial communities was, indeed, significantly different from gut microbiota, resulting mainly constituted of cyanobacteria, Proteobacteria, and Firmicutes phyla. According to PERMANOVA analysis, unweighted UniFrac indicated that either the diet or the host genotype plays a crucial role in shaping the gut microbiome, showing differences between the two strains fed the same diet and an overall effect of diet for ITA strain only. This result could explain the differences observed in growth performance between the two strains, with the United States trout growing more efficiently than the ITA fish when fed either the low-FM or the all-plant protein diets.

The current results seemed to indicate an existence of a relationship between the selected United States strain and the *Cetobacterium* genus, belonging to the Fusobacteriaceae family, whose relative abundance was an order of magnitude larger than that detected in the ITA strain. Similarly, higher abundances of the phyla Bacteroidetes and Fusobacteria phyla are associated with trout selected for high-muscle yield (Chapagain et al., 2020). Fusobacteria is commonly found in the freshwater fish guts, and *Cetobacterium* is an important commensal bacterium (Navarrete et al., 2012). Metabolites produced by *Cetobacterium* include, indeed, short-chain fatty acids (SCFAs), such as acetate, propionate, and butyrate, and vitamin B12, which can enhance fish health (Xie et al., 2021).

The *in silico* prediction of the metabolic capability of the bacterial populations revealed an up-regulation of the pathway related to starch and sucrose metabolism in the United States strain fed the C diet, thus indicating a better ability to utilize carbohydrates. This is not surprising, as the United States strain has been selected for its growth performance when fed all-plant protein feeds (rich in fibers and non-digestible carbohydrates). The fatty acid metabolism pathway has previously been reported to be enriched in high-muscle yield selected trout (Chapagain et al., 2020). However, despite the different pathways being activated, an up-regulation of bacterial pathways involved in energy supply–which is essential for host growth–was herein analogously observed.

## Conclusion

In conclusion, the selected strain of rainbow trout overall displayed improved growth performance and (to a lesser extent) whole-body proximate composition when compared to the non-selected one. However, the absence of negative changes in the non-selected strain in response to all-plant protein feed is indicative of a proper adaptation of the fish to such protein sources over time, thus suggesting that a complete FM replacement in diets for rainbow trout is technically feasible. Furthermore, differences between microbial communities were mainly driven by host genetic selection, with the *Cetobacterium* probiotic-like genus being associated with the gut of the selected rainbow trout. Such microbiota signature, along with the up-regulation of the pathway involved in starch and sucrose metabolism, may reasonably predispose the selected strain to efficiently utilize all-plant protein feeds. Future research assessing additional parameters related to the gut health of the fish (i.e., gut mucosa morphology, mucin dynamics, inflammatory response, and metabolomics) are strongly recommended to further investigate the role of genetic selection in the digestion of all-plant protein diets.

## Data Availability

All fastq sequencing files were deposited in the EBI ENA public database under the accession project code PRJEB51166.
