# Supplementary material for: Efficacy of Utilization of All-Plant-Based and Commercial Low-Fishmeal Feeds in Two Divergently Selected Strains of Rainbow Trout (Oncorhynchus mykiss): Focus on Growth Performance, Whole-Body Proximate Composition, and Intestinal Microbiome
Source: Front Physiol. 2022 May 20;13:892550. doi: 10.3389/fphys.2022.892550 (PMC9163680; doi:10.3389/fphys.2022.892550)
Supplement: Supplementary file 1 [file Table1.docx]

**Supplementary Table S1.** The list of the most abundant bacterial taxa, their relative abundance

values, and statistical analysis.

| **Taxa** | **Feed_C** | | | **Feed_PP** | | | **P-value** |
| --- | --- | --- | --- | --- | --- | --- | --- |
|  |  |  |  |  |  |  |  |
| **Phylum** |  |  |  |  |  |  |  |
| Cyanobacteria | 82.23 | ± | 0.19 | 81.37 | ± | 1.90 | 0.571 |
| Firmicutes | 4.33 | ± | 0.09 | 4.94 | ± | 1.66 | 0.688 |
| Proteobacteria | 13.42 | ± | 0.12 | 13.21 | ± | 0.43 | 0.827 |
| **Class** |  |  |  |  |  |  |  |
| Chloroplast | 82.23 | ± | 0.23 | 81.36 | ± | 2.33 | 0.565 |
| Bacilli | 4.24 | ± | 0.16 | 4.94 | ± | 2.04 | 0.636 |
| Alphaproteobacteria | 11.65 | ± | 0.16 | 11.70 | ± | 0.60 | 0.909 |
| Gammaproteobacteria | 1.76 | ± | 0.03 | 1.31 | ± | 0.74 | 0.305 |
| **Order** |  |  |  |  |  |  |  |
| Streptophyta | 82.23 | ± | 0.23 | 81.36 | ± | 2.33 | 0.565 |
| Bacillales | 0.18 | ± | 0.09 | 2.04 | ± | 2.27 | 0.158 |
| Lactobacillales | 4.06 | ± | 0.26 | 2.90 | ± | 0.32 | **0.009** |
| Rickettsiales | 11.65 | ± | 0.16 | 11.70 | ± | 0.60 | 0.909 |
| Pseudomonadales | 1.15 | ± | 0.05 | 0.89 | ± | 0.58 | 0.407 |
| **Family** |  |  |  |  |  |  |  |
| Planococcaceae | 0.14 | ± | 0.08 | 1.86 | ± | 2.21 | 0.182 |
| Lactobacillaceae | 0.06 | ± | 0.04 | 0.18 | ± | 0.11 | 0.145 |
| Leuconostocaceae | 1.15 | ± | 0.05 | 2.15 | ± | 0.35 | **0.005** |
| Streptococcaceae | 2.13 | ± | 0.27 | 0.53 | ± | 0.06 | **0.049** |
| Moraxellaceae | 11.65 | ± | 0.16 | 11.70 | ± | 0.60 | 0.909 |
| Enterococcaceae | 0.20 | ± | 0.02 | 0.00 | ± | 0.00 | **0.046** |
| Aeromonadaceae | 0.19 | ± | 0.05 | 0.01 | ± | 0.01 | **0.001** |
| Shewanellaceae | 0.17 | ± | 0.03 | 0.40 | ± | 0.16 | **0.048** |
| Enterobacteriaceae | 0.88 | ± | 0.07 | 0.68 | ± | 0.49 | 0.432 |
| Pseudomonadaceae | 0.27 | ± | 0.02 | 0.21 | ± | 0.09 | 0.323 |
| Oxalobacteraceae | 0.01 | ± | 0.01 | 0.20 | ± | 0.05 | **0.002** |
| Fusobacteriaceae | 0.00 | ± | 0.00 | 0.20 | ± | 0.10 |  |
| Mycoplasmataceae | 0.00 | ± | 0.00 | 0.24 | ± | 0.22 |  |
| **Genus** |  |  |  |  |  |  |  |
| Enterococcus | 0.03 | ± | 0.05 | 0.18 | ± | 0.11 | 0.078 |
| Lactobacillus | 0.66 | ± | 0.01 | 2.08 | ± | 0.35 | **0.049** |
| Pediococcus | 0.41 | ± | 0.04 | 0.04 | ± | 0.02 | **0.000** |
| Leuconostoc | 0.77 | ± | 0.07 | 0.07 | ± | 0.01 | **0.049** |
| Weissella | 1.36 | ± | 0.23 | 0.46 | ± | 0.07 | **0.001** |
| Lactococcus | 0.66 | ± | 0.01 | 0.04 | ± | 0.02 | **0.049** |
| Raphanus | 0.32 | ± | 0.07 | 0.01 | ± | 0.01 | **0.001** |
| Aeromonas | 0.20 | ± | 0.02 | 0.00 | ± | 0.00 | **0.046** |
| Shewanella | 0.19 | ± | 0.05 | 0.01 | ± | 0.01 | **0.001** |
| Acinetobacter | 0.88 | ± | 0.07 | 0.25 | ± | 0.04 | **0.000** |
| Pseudomonas | 0.27 | ± | 0.02 | 0.21 | ± | 0.09 | 0.324 |
| Massilia | 0.01 | ± | 0.01 | 0.20 | ± | 0.05 | **0.002** |
| Cetobacterium | 0.00 | ± | 0.00 | 0.20 | ± | 0.10 |  |
| Enhydrobacter | 0.00 | ± | 0.00 | 0.43 | ± | 0.47 |  |
