# Supplementary material for: Efficacy of Utilization of All-Plant-Based and Commercial Low-Fishmeal Feeds in Two Divergently Selected Strains of Rainbow Trout (Oncorhynchus mykiss): Focus on Growth Performance, Whole-Body Proximate Composition, and Intestinal Microbiome
Source: Front Physiol. 2022 May 20;13:892550. doi: 10.3389/fphys.2022.892550 (PMC9163680; doi:10.3389/fphys.2022.892550)
Supplement: Supplementary file 2 [file Table2.docx]

**Supplementary Table S2.** The most abundant bacterial taxa with their relative abundance data and statistical analysis.

| **Taxa** | **ITA_C** | | | **ITA_PP** | | | **USA_C** | | | **USA_PP** | | | ***Sig.*** | | |
| --- | --- | --- | --- | --- | --- | --- | --- | --- | --- | --- | --- | --- | --- | --- | --- |
|  |  |  |  |  |  |  |  |  |  |  |  |  | **Diet** | **Strain** | **Interaction** |
| **Phylum** |  |  |  |  |  |  |  |  |  |  |  |  |  |  |  |
| Tenericutes | 97.25 | ± | 4.55 | 99.21 | ± | 1.68 | 93.56 | ± | 10.96 | 93.43 | ± | 12.89 | 0.779 | 0.181 | 0.687 |
| Proteobacteria | 2.56 | ± | 4.28 | 0.39 | ± | 0.87 | 0.75 | ± | 1.37 | 5.27 | ± | 12.13 | 0.940 | 0.579 | 0.255 |
| Fusobacteriota | 0.05 | ± | 0.09 | 0.40 | ± | 0.81 | 5.69 | ± | 9.61 | 1.24 | ± | 1.32 | 0.701 | 0.049 | 0.238 |
| Class |  |  |  |  |  |  |  |  |  |  |  |  |  |  |  |
| Mollicutes | 97.25 | ± | 4.55 | 99.21 | ± | 1.68 | 93.56 | ± | 10.96 | 93.43 | ± | 12.89 | 0.779 | 0.181 | 0.687 |
| Gammaproteobacteria | 2.51 | ± | 4.17 | 0.39 | ± | 0.87 | 0.75 | ± | 1.37 | 5.27 | ± | 12.13 | 0.931 | 0.566 | 0.258 |
| Fusobacteriia | 0.05 | ± | 0.09 | 0.40 | ± | 0.81 | 5.69 | ± | 9.61 | 1.24 | ± | 1.32 | 0.701 | **0.049** | 0.238 |
| **Order** |  |  |  |  |  |  |  |  |  |  |  |  |  |  |  |
| Enterobacterales | 0.08 | ± | 0.11 | 0.23 | ± | 0.55 | 0.06 | ± | 0.12 | 2.83 | ± | 6.48 | 0.244 | 0.299 | 0.283 |
| Pseudomonadales | 0.99 | ± | 2.40 | 0.00 | ± | 0.00 | 0.00 | ± | 0.00 | 0.01 | ± | 0.02 | 0.255 | 0.296 | 0.251 |
| Burkholderiales | 1.32 | ± | 2.08 | 0.11 | ± | 0.21 | 0.65 | ± | 1.25 | 2.37 | ± | 5.52 | 0.901 | 0.524 | 0.338 |
| Mycoplasmatales | 97.33 | ± | 4.45 | 99.21 | ± | 1.68 | 93.56 | ± | 10.96 | 93.49 | ± | 12.92 | 0.794 | 0.185 | 0.756 |
| Fusobacteriales | 0.05 | ± | 0.09 | 0.40 | ± | 0.81 | 5.69 | ± | 9.61 | 1.24 | ± | 1.32 | 0.701 | **0.049** | 0.238 |
| **Family** |  |  |  |  |  |  |  |  |  |  |  |  |  |  |  |
| Aeromonadaceae | 0.13 | ± | 0.31 | 0.00 | ± | 0.00 | 0.01 | ± | 0.03 | 0.05 | ± | 0.13 | 0.497 | 0.715 | 0.273 |
| Moraxellaceae | 0.99 | ± | 2.40 | 0.00 | ± | 0.00 | 0.00 | ± | 0.00 | 0.01 | ± | 0.02 | 0.255 | 0.296 | 0.251 |
| Mycoplasmataceae | 97.33 | ± | 4.45 | 99.21 | ± | 1.68 | 93.56 | ± | 10.96 | 93.49 | ± | 12.92 | 0.794 | 0.185 | 0.756 |
| Fusobacteriaceae | 0.05 | ± | 0.09 | 0.40 | ± | 0.81 | 5.69 | ± | 9.61 | 1.24 | ± | 1.32 | 0.701 | **0.049** | 0.238 |
| Chitinibacteraceae | 0.62 | ± | 1.01 | 0.10 | ± | 0.19 | 0.27 | ± | 0.54 | 2.17 | ± | 5.13 | 0.753 | 0.504 | 0.325 |
| Chromobacteriaceae | 0.70 | ± | 1.30 | 0.01 | ± | 0.02 | 0.38 | ± | 0.72 | 0.20 | ± | 0.40 | 0.223 | 0.645 | 0.433 |
| Yersiniaceae | 0.00 | ± | 0.00 | 0.09 | ± | 0.22 | 0.01 | ± | 0.01 | 1.15 | ± | 2.65 | 0.127 | 0.225 | 0.335 |
| **Genus** |  |  |  |  |  |  |  |  |  |  |  |  |  |  |  |
| Aeromonas | 0.13 | ± | 0.31 | 0.00 | ± | 0.00 | 0.02 | ± | 0.03 | 0.06 | ± | 0.14 | 0.501 | 0.861 | 0.391 |
| Mycoplasma | 97.33 | ± | 4.45 | 99.34 | ± | 1.36 | 93.57 | ± | 10.95 | 94.75 | ± | 10.09 | 0.672 | 0.190 | 0.811 |
| Cetobacterium | 0.05 | ± | 0.09 | 0.40 | ± | 0.82 | 5.69 | ± | 9.62 | 1.29 | ± | 1.39 | 0.715 | **0.048** | 0.246 |
| Enhydrobacter | 0.62 | ± | 1.01 | 0.00 | ± | 0.00 | 0.00 | ± | 0.00 | 0.01 | ± | 0.02 | 0.263 | 0.335 | 0.257 |
| Deefgea | 0.70 | ± | 1.30 | 0.10 | ± | 0.20 | 0.27 | ± | 0.54 | 2.39 | ± | 5.67 | 0.729 | 0.491 | 0.324 |
| Crenobacter | 0.00 | ± | 0.00 | 0.01 | ± | 0.02 | 0.38 | ± | 0.72 | 0.22 | ± | 0.44 | 0.234 | 0.632 | 0.425 |

Abbreviations: ITA, Italian strain; USA, American strain; C, commercial diet; PP1, all-plant protein diet.
