# Supplementary figures and images for: Efficacy of Utilization of All-Plant-Based and Commercial Low-Fishmeal Feeds in Two Divergently Selected Strains of Rainbow Trout (Oncorhynchus mykiss): Focus on Growth Performance, Whole-Body Proximate Composition, and Intestinal Microbiome
Source: Front Physiol. 2022 May 20;13:892550. doi: 10.3389/fphys.2022.892550 (PMC9163680; doi:10.3389/fphys.2022.892550)

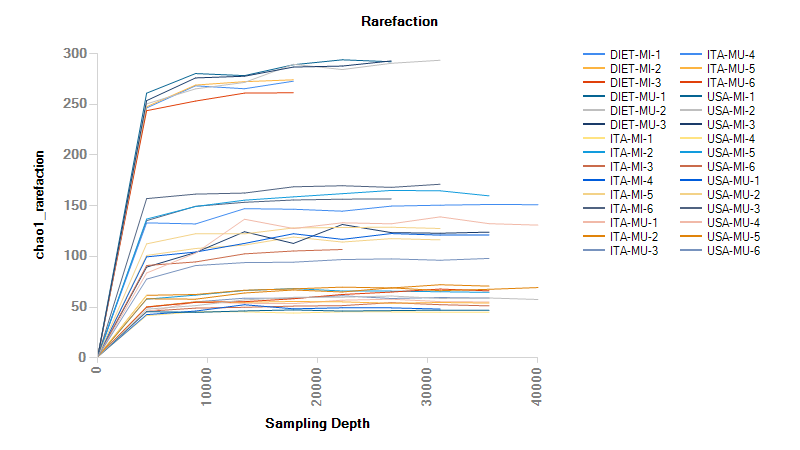

Supplement: Supplementary file 3 [file Image1.png]
